# Dynamics of *DNMT3A* mutation and prognostic relevance in patients with primary myelodysplastic syndrome

**DOI:** 10.1186/s13148-018-0476-1

**Published:** 2018-04-02

**Authors:** Ming-En Lin, Hsin-An Hou, Cheng-Hong Tsai, Shang-Ju Wu, Yuan-Yeh Kuo, Mei-Hsuan Tseng, Ming-Chih Liu, Chia-Wen Liu, Wen-Chien Chou, Chien-Yuan Chen, Jih-Luh Tang, Ming Yao, Chi-Cheng Li, Shang-Yi Huang, Bor-Sheng Ko, Szu-Chun Hsu, Chien-Ting Lin, Hwei-Fang Tien

**Affiliations:** 1 Division of Hematology, Department of Internal Medicine, National Taiwan University Hospital, No.7, Chung Shan S. Rd., Zhongzheng Dist, Taipei, 10002 Taiwan; 2 Division of Hematology, Department of Internal Medicine, National Taiwan University Hospital, Hsin-Chu Branch, Hsinchu City, Taiwan; 3 Graduate Institute of Clinical Medicine, College of Medicine, National Taiwan University, Taipei, Taiwan; 4 Tai-Cheng Stem Cell Therapy Center, National Taiwan University, Taipei, Taiwan; 5 Graduate Institute of Oncology, College of Medicine, National Taiwan University, Taipei, Taiwan; 6 Departments of Pathology, National Taiwan University Hospital, Taipei, Taiwan; 7 Department of Laboratory Medicine, National Taiwan University Hospital, Taipei, Taiwan

**Keywords:** *DNMT3A*, Myelodysplastic syndrome, Prognosis, Paired samples

## Abstract

**Background:**

*DNMT3A* gene mutation has been associated with poor prognosis in acute myeloid leukemia, but its clinical implications in myelodysplastic syndrome (MDS) and dynamic changes during disease progression remain controversial.

**Results:**

In this study, *DNMT3A* mutation was identified in 7.9% of 469 de novo MDS patients. *DNMT3A*-mutated patients had higher platelet counts at diagnosis, and patients with ring sideroblasts had the highest incidence of *DNMT3A* mutations, whereas those with multilineage dysplasia had the lowest incidence. Thirty-one (83.8%) of 37 *DNMT3A*-mutated patients had additional molecular abnormalities at diagnosis, and *DNMT3A* mutation was highly associated with mutations of *IDH2* and *SF3B1*. Patients with *DNMT3A* mutations had a higher risk of leukemia transformation and shorter overall survival. Further, *DNMT3A* mutation was an independent poor prognostic factor irrespective of age, IPSS-R, and genetic alterations. The sequential study demonstrated that the original *DNMT3A* mutations were retained during follow-ups unless allogeneic hematopoietic stem cell transplantation was performed, while *DNMT3A* mutation was rarely acquired during disease progression.

**Conclusions:**

*DNMT3A* mutation predicts unfavorable outcomes in MDS and was stable during disease evolutions. It may thus be a potential biomarker to predict prognosis and monitor the treatment response.

**Electronic supplementary material:**

The online version of this article (10.1186/s13148-018-0476-1) contains supplementary material, which is available to authorized users.

## Background

Alterations of epigenetic regulation may result in aberrations of gene expression and malignant transformation of cells [1, 2]. DNA methylation, one of the mechanisms for epigenetic control of gene expression, regulates important physiological development, such as gene imprint and X-chromosome inactivation [3, 4]. In mammalians, three DNA methyltransferase (DNMT), namely DNMT1, 3A, and 3B have been identified [5]. Mutation of *DNMT3A* gene has been reported in patients with myeloid malignancies, including myelodysplastic syndromes (MDS) and acute myeloid leukemia (AML) [6–13].

MDS represent a clinically heterogeneous hematologic neoplasm characterized by variable degrees of cytopenias and risk of leukemia transformation [14]. The incidence (2.6 to 20.2%) of *DNMT3A* mutation in MDS varied widely, possibly due to different patient population and methods used [12, 13, 15, 16]. Regarding the prognostic relevance, *DNMT3A* mutation has been reported to predict poor prognosis in AML patients [7–11]. However, the prognostic implications of *DNMT3A* mutation in MDS are still controversial [12, 13, 15, 17]. Walter et al. [12] and Thol et al. [13] reported that *DNMT3A* mutation was associated with higher risk of leukemia transformation and shorter survival, but the other studies failed to find these associations [15, 17]. Besides, sequential studies to evaluate the dynamic changes of *DNMT3A* mutations during disease evolution in MDS are limited. In the present study, we investigated the *DNMT3A* mutation in 469 patients with de novo MDS and analyzed its associations with the clinical characteristics, outcomes, and other genetic alterations. We also performed sequential analysis of the *DNMT3A* gene mutation for 431 samples from 148 patients to evaluate the stability of *DNMT3A* mutation during the clinical course.

## Methods

### Subjects

This study was approved by the Institutional Review Board/Ethical Committee of the National Taiwan University Hospital (NTUH). Diagnosis and classification of MDS were made according to the French-American-British (FAB) Cooperative Group Criteria and the WHO 2016 classification [18, 19]. From May 1985 to December 2010, a total of 469 adult patients with newly diagnosed MDS at the NTUH who had enough cryopreserved cells for analysis were enrolled. Patients with secondary or therapy-related MDS were excluded. The disease of 362 patients fulfilled the criteria of MDS according to the 2016 WHO classification. Most patients (77.4%) received only palliative treatment, including transfusions, hematopoietic growth factors, immunosuppressive therapy, and low-intensity chemotherapy. Thirty (6.4%) patients received intensive chemotherapy, 7.2% received hypomethylating agents (HMA), and 9.0% received allogeneic hematopoietic stem cell transplantation (HSCT).

### Analyses of mutations

Mutational analysis of *DNMT3*A gene exons 2-23 by PCR and direct sequencing was done as described previously [9]. Analysis of the mutations in other genes involving in activated signaling pathways, such as *FLT3*-ITD [20], *NRAS* [21], *KRAS* [21], *JAK2* [21], and *PTPN11* [22]; the transcription factor, such as *RUNX1* [23]; splicing factors, including *SRSF2*, *U2AF1*, and *SF3B1* [24]; and epigenetic modifications, including *MLL/*PTD [25], *ASXL1* [26], *EZH2* [27], *IDH1* [28], *IDH2* [29], and *TET2* [30], as well as *SETBP1* [21], *WT1* [31], *NPM1* [32], and *TP53* [33], were performed as previously described. To detect *DNMT3A* mutation, we used DNA amplified in vitro from bone marrow (BM) cells with the Illustra GenomiPhi V2 DNA-amplification kit (GE Healthcare, UK). All mutations detected were verified in the original non-amplified samples [34]. Abnormal sequencing results were confirmed by at least two repeated analyses. All nonsense or frameshift mutations were regarded as true mutations. Missense mutations were regarded as true only if they were documented in other studies or could be verified by sequencing of matched normal somatic tissues. Serial analyses of *DNMT3*A mutations during the clinical course were also performed in 431 samples from 148 patients.

### TA cloning analysis

For the patients with discrepancy of the mutation status of the *DNMT3A* in sequential samples, TA cloning was performed in the samples without detectable mutation followed by direct sequencing. More than 30 clones were selected for sequencing as previously described [9].

### Illumina next generation sequencing (NGS) for serial studies of patients with *DNMT3A* mutation

Serial analyses of mutations at diagnosis, disease progression, and/or remission were further performed using Illumina next generation sequencing in 32 samples from 13 patients with *DNMT3A* mutation at diagnosis and one during follow-up. Genomic DNA extracted from BM mononuclear cells was analyzed for mutations in 54 genes involved in myeloid malignancies by TruSight Myeloid Panel (Illumina, San Diego, CA, USA). HiSeq platform (Illumina) was used for sequencing with a median reading depth of 12,000×.

### Statistical analysis

The discrete variables of patients with and without gene mutations were compared using the *χ*^2^ tests, and the Fisher’s exact test was used if the expected values of contingency tables were smaller than 5. The continuous variables of patients with and without gene mutations were compared using Student’s *t* test. If the data were not normally distributed, Mann-Whitney *U* tests were used to compare continuous variables and medians of distributions. Overall survival (OS) was measured from the date of first diagnosis to the date of last follow-up or death from any cause. Time to leukemia transformation was measured from the date of MDS diagnosis to the date confirmed of acute leukemic change. Kaplan-Meier estimation was used to plot survival curves, and log-rank tests were used to calculate the difference of OS and time to leukemia transformation between groups. Multivariate Cox proportional hazard regression analysis was used to investigate independent prognostic factors for OS and time to leukemia transformation. All tests were 2-tailed, and *P* < 0.05 was considered statistically significant. All statistical analyses were performed with SPSS Version 17 software.

## Results

### *DNMT3A* mutations in patients with de novo MDS

**Fig. 1:**
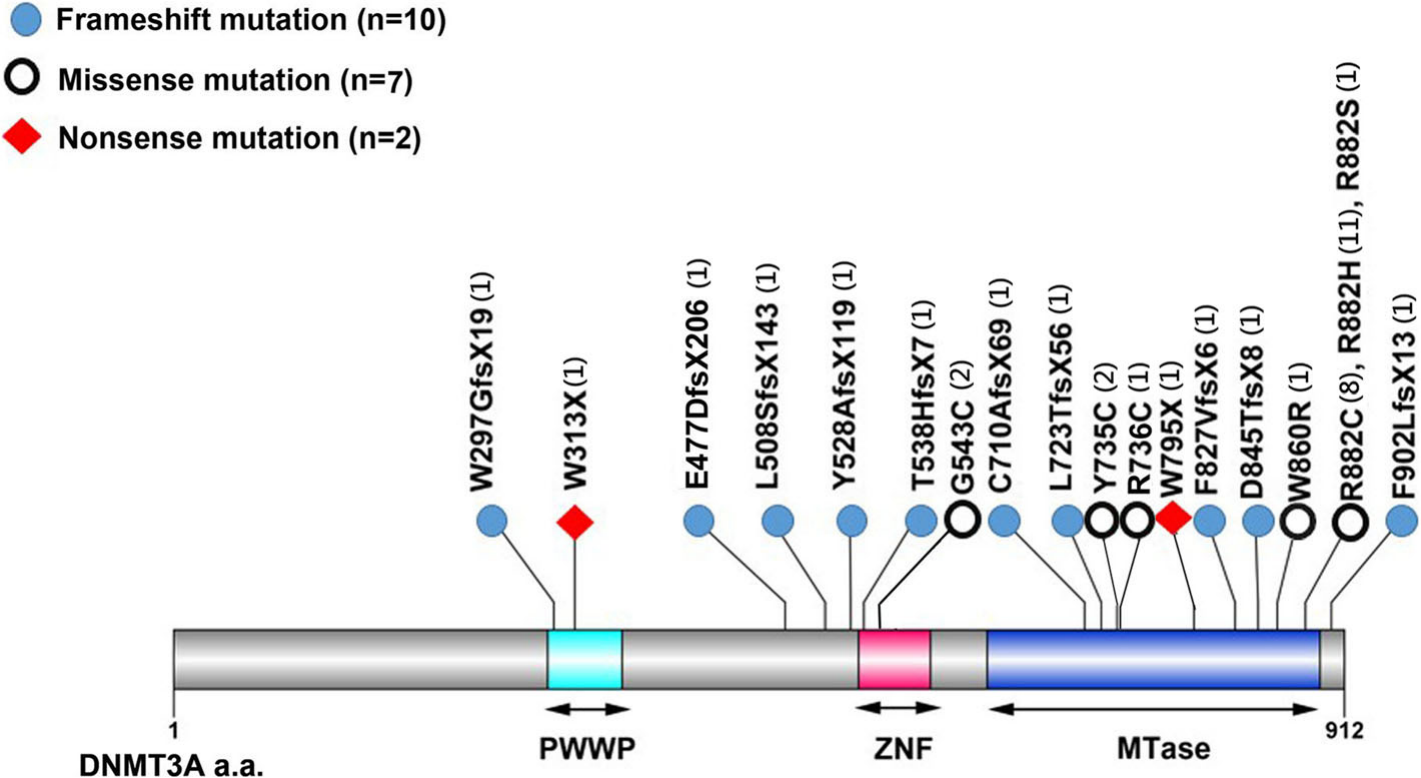
Patterns and locations of *DNMT3A* mutations

A total of 469 patients with de novo MDS according to the FAB classification were included for mutational analysis. Among them, 171 (36.5%) patients had refractory anemia (RA), 32 (6.8%) had RA with ring sideroblasts (RARS), 159 (33.9%) had RA with excess blasts (RAEB), 53 (11.3%) had RAEB in transformation (RAEB-T), and 54 (11.5%) had chronic myelomonocytic leukemia (CMML) (Table 1). Nineteen different *DNMT3A* mutations were identified in 37 (7.9%) of the 469 patients, including 7 missense mutations, 2 nonsense mutations, and 10 frameshift mutations (Fig. 1). In addition to the 13 single-nucleotide polymorphisms without amino acid residue alterations, 9 missense mutations with uncertain biologic significance were excluded because they were not reported previously and could not be verified for lack of matched normal somatic tissues or remission BM samples. Thirty-six of the 37 *DNMT3A*-mutated patients had single heterozygous mutation, and the remaining one patient had double mutations. The most common mutation was R882H (*n* = 11), followed by R882C (*n* = 8), G543C (*n* = 2), and Y735C (*n* = 2). All other mutations were detected in only one patient each (Fig. 1; Additional file 1: Table S1). According to the 2016 WHO classification, *DNMT3A* mutations were identified in 28 (7.7%) of the 362 patients (Table 1).

**Table 1:** Comparison of clinical features between MDS patients with and without *DNMT3A* mutation

Variables	Total	*DNMT3A* mutated	*DNMT3A* wild	*P* value
	(*n* = 469)	(*n* = 37, 7.9%)	(*n* = 432, 92.1%)	
Gender^†^				0.201
Male	315 (67.2)	21 (6.7)	294 (93.3)	
Female	154 (32.8)	16 (10.4)	138 (89.6)	
Age (year)^#^	65.5 (16~98)	69.6 (35~89)	65.5 (16~98)	0.151
Lab data^#^				
WBC (× 10^9^/L)	3.84 (0.44~355.3)	5.02 (0.49~59.83)	3.73 (0.44~355.3)	0.942
Hb (g/dL)	8.3 (3~15)	8.1 (5~11)	8.3 (3~15)	0.555
Platelet (× 10^9^/L)	74 (2~931)	162 (14~460)	74 (2~931)	0.045
LDH (mckat/L)	8.133 (2.422~113.677)	8.208 (3.507~24.733)	8.133 (2.422~113.677)	0.739
FAB subtype^††^				0.002
RA	171 (36.5)	4 (2.3)	167 (97.7)	0.001
RARS	32 (6.8)	6 (18.8)	26 (81.2)	0.031
RAEB	159 (33.9)	18 (11.3)	141 (88.7)	0.069
RAEB-T	53 (11.3)	5 (9.4)	48 (90.6)	0.593
CMML	54 (11.5)	4 (7.4)	50 (92.6)	> 0.999
2016 WHO classification ^††^	(*n* = 362)	(*n* = 28, 7.7%)	(*n* = 334, 92.3%)	0.011
MDS-SLD	60 (16.6)	1 (1.7)	59 (98.3)	0.062
MDS-MLD	106 (29.3)	3 (2.8)	103 (97.2)	0.029
MDS-RS-SLD	18 (5.0)	3 (16.7)	15 (83.3)	0.154
MDS-RS-MLD	13 (3.6)	3 (23.1)	10 (76.9)	0.070
MDS with isolated del(5q)	2 (0.6)	0 (0.0)	2 (100.0)	> 0.999
MDS-EB-1	78 (21.5)	8 (10.3)	70 (89.7)	0.343
MDS-EB-2	81 (22.4)	10 (12.3)	71 (87.7)	0.097
MDS-U	4 (1.1)	0 (0.0)	4 (100.0)	> 0.999
Karyotype risk^†† Φ^	(*n* = 437)	(*n* = 35)	(*n* = 402)	0.883
Good	263 (60.2)	20 (7.6)	243 (92.4)	0.721
Intermediate	88 (20.1)	7 (8.0)	81 (92.0)	> 0.999
Poor	86 (19.7)	8 (9.3)	78 (90.7)	0.658
IPSS^††^ §	(*n* = 437)	(*n* = 35)	(*n* = 402)	0.372
Low	68 (15.6)	4 (5.9)	64 (94.1)	0.630
INT-1	181 (41.4)	11 (6.0)	170 (94.0)	0.283
INT-2	107 (24.5)	11 (10.3)	96 (89.7)	0.412
High	81 (18.5)	9 (11.1)	72 (88.9)	0.259
IPSS-R^††^ζ	(*n* = 437)	(*n* = 35)	(*n* = 402)	0.692
Very low	13 (3.0)	1 (7.7)	12 (99.3)	> 0.999
Low	104 (23.8)	7 (6.7)	97 (93.3)	0.682
Intermediate	106 (24.2)	6 (5.7)	100 (94.3)	0.411
High	113 (25.9)	10 (8.8)	103 (91.2)	0.841
Very high	101 (23.1)	11 (10.9)	90 (89.1)	0.294

*Abbreviations*: *FAB*, French-American-British classification; *RA*, refractory anemia; *RARS*, refractory anemia with ring sideroblasts; *RAEB*, refractory anemia with excess blasts; *RAEB-T*, refractory anemia with excess blasts in transformation; *CMML*, chronic myelomonocytic leukemia; *MDS-SLD*, MDS with single lineage dysplasia; *MDS-MLD*, MDS with multilineage dysplasia; *MDS-RS-SLD*, MDS with ring sideroblasts with single lineage dysplasia; *MDS-RS-MLD*, MDS with ring sideroblasts with multilineage dysplasia; *MDS-EB1* , MDS with excess blasts-1; *MDS-EB2*, MDS with excess blasts-2; *MDS-U*, MDS, unclassified

^†^Number of patients (% among the males or females)

^#^Median (range)

^††^Number of patients (% among patients of each subgroup)

^Φ^Good: normal karyotype, isolated -Y, del(5q), or del(20q); Poor: complex (≧ 3 abnormalities) or chromosome 7 anomalies; Intermediate, other abnormalities

§ IPSS (international prognosis scoring system): low, 0; intermediate (INT)-1, 0.5–1; INT-2, 1.5–2; and high, ≥ 2.5

ζ IPSS-R (revised international prognostic scoring system): very low, ≦1.5; low, > 1.5–3; intermediate, > 3–4.5; high, > 4.5–6; and very high, > 6

### Correlation of *DNMT3A* mutations with clinical features

*DNMT3A*-mutated patients had higher platelet counts at diagnosis than *DNMT3A*-wild patients (Table 1). According to the FAB classification, *DNMT3A* mutation was highly associated with RARS subtype. Patients with RARS had the highest incidence (18.8%, *P* = 0.031) of *DNMT3A* mutations, whereas those with RA had the lowest incidence (2.3%, *P* = 0.001). By the 2016 WHO classification, patients with MDS with multilineage dysplasia (MDS-MLD) had lower incidence of *DNMT3A* mutations (2.8%, *P* = 0.029). No association between age or gender of patients and *DNMT3A* mutation status was found (Table 1). There was also no difference in the distribution of risk groups according to the international prognostic scoring system (IPSS) or revised IPSS (IPSS-R) between patients with and without *DNMT3A* mutations (Table 1).

Chromosome data were available in 437 (93.2%) patients at diagnosis, and clonal chromosomal abnormalities were detected in 193 (44.2%) patients. There were no association of the *DNMT3A* mutations with common chromosomal abnormalities, including loss of Y, − 20/del(20q), − 5/del(5q), + 8, and − 7/del(7q) (Additional file 1: Table S2), or risks of karyotype (Table 1).

### Association of *DNMT3A* mutation with other genetic mutations

Among the 37 patients with *DNMT3A* mutations, 33 (89.2%) patients had additional molecular abnormalities at diagnosis, including *SF3B1* (*n* = 11), *TET2* (*n* = 7), *IDH2* (*n* = 7), *RUNX1* (*n* = 7), *ASXL1* (*n* = 5), *SRSF2* (*n* = 5), *TP53* (*n* = 4), *N-RAS* (*n* = 2), *MLL/*PTD (*n* = 2), *U2AF1* (*n* = 2), *NPM1* (*n* = 2), *K-RAS* (*n* = *1*), and *IDH1* mutations (*n* = 1) (Additional file 1: Table S1). Fifteen patients had 1 additional mutation, 13 had 2, and 5 had 3 (Additional file 1: Table S1). Patients with *DNMT3A* mutations had a significantly higher incidence of *SF3B1* and *IDH2* mutations than those without *DNMT3A* mutations (*P* < 0.001 and *P* < 0.001, respectively; Table 2).

**Table 2:** Comparison of other genetic alterations between MDS patients with and without the *DNMT3A* mutation

		Number and percentage of patients with the mutation (%)			
Mutation	No. examined	Total patients	*DNMT3A*-mutated patients	*DNMT3A*-wild patients	*P* value
*IDH1*	468	4 (0.9)	1 (2.7)	3 (0.7)	0.281
*IDH2*	464	19 (4.1)	7 (18.9)	12 (2.8)	< 0.001
*ASXL1*	459	108 (23.5)	5 (13.5)	103 (24.4)	0.160
*EZH2*	469	29 (6.2)	0 (0.0)	29 (6.7)	0.153
*TET2*	469	61 (13.0)	7 (18.9)	54 (12.5)	0.304
*FLT3/*ITD	465	5 (1.1)	0 (0)	5 (1.2)	> 0.999
*JAK2*	467	4 (0.9)	0 (0.0)	4 (0.9)	> 0.999
*NRAS*	469	25 (5.3)	2 (5.4)	23 (5.3)	> 0.999
*KRAS*	465	8 (1.7)	1 (2.7)	7 (1.6)	0.488
*PTPN11*	119	1 (0.8)	0 (0)	1 (1.0)	> 0.999
*WT1*	256	1 (0.4)	0 (0)	1 (0.4)	> 0.999
*MLL/*PTD	447	5 (1.1)	2 (5.4)	3 (0.7)	0.057
*RUNX1*	462	61 (13.2)	7 (18.9)	54 (12.7)	0.308
*U2AF1*	469	35 (7.5)	2 (5.4)	33 (7.6)	> 0.999
*SRSF2*	469	60 (12.8)	5 (13.5)	55 (12.7)	0.801
*SF3B1*	469^#^	48 (10.2)	11 (29.7)	37 (8.6)	< 0.001
*Lower-risk IPSS*	249	33 (13.3)	7 (46.7)	26 (11.1)	0.001
*Higher-risk IPSS*	188	11 (5.9)	3 (15)	8 (4.8)	0.098
*SETBP1*	466	15 (3.2)	0 (0)	15 (3.5)	0.621
*TP53*	465	42 (9.0)	4 (10.8)	38 (8.9)	0.763

*Abbreviations: No.* number, *ITD* internal tandem duplication, *PTD* partial tandem duplication

^#^Four hundred and thirty-seven of them had cytogenetic data and could be assigned to the IPSS-R risk groups

### Correlation of *DNMT3A* mutation with clinical outcome

**Fig. 2:**
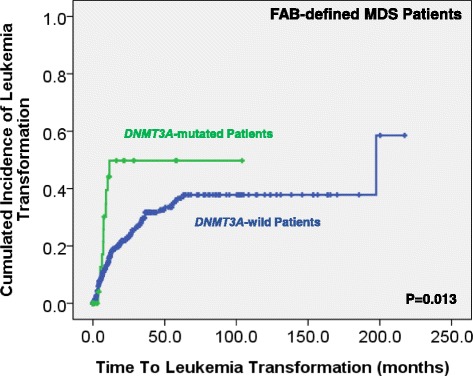
Kaplan-Meier curves stratified by the status of *DNMT3A* mutations for time to leukemia transformation among the whole cohort of 469 MDS patients according to the FAB classification

**Fig. 3:**
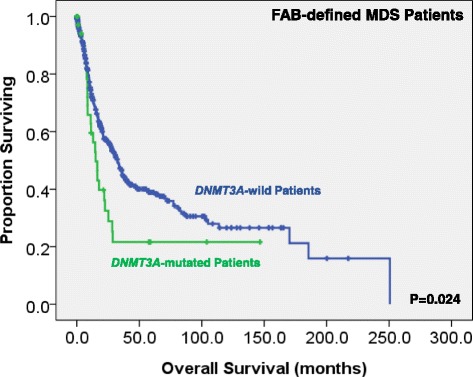
Kaplan-Meier curves stratified by the status of *DNMT3A* mutations for overall survival among the whole cohort of 469 MDS patients according to the FAB classification

**Fig. 4:**
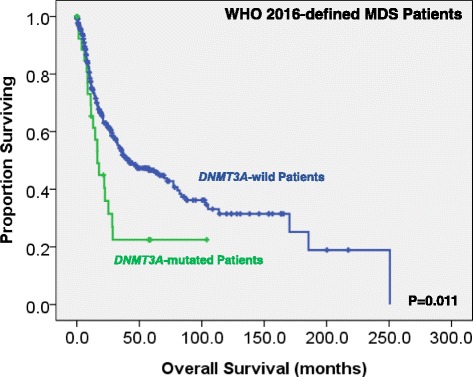
Kaplan-Meier curves stratified by the status of *DNMT3A* mutations for overall survival among 362 patients based on the 2016 WHO classification

We could not find the difference in treatment regimens between the patients with *DNMT3A* mutations and those without. With a median follow-up of 43.9 months (range 0.1–250.7 months), patients with *DNMT3A* mutations had a higher risk to transform to AML (5-year AML transformation rate, 34.4 versus 22.5%, *P* = 0.013; Fig. 2). MDS patients, based on either the FAB or the 2016 WHO classification, had a significantly shorter OS if they harbored *DNMT3A* mutation than those who did not (15.0 versus 32.5 months, *P* = 0.024, and 16.3 versus 41.6 months, *P* = 0.011, respectively; Figs. 3 and 4). Further, we could not find the survival difference between the patients with frameshift and non-frameshift mutations. Interestingly, patients with *DNMT3A* mutations had a better OS if they received allogenic HSCT than those who did not (*P* = 0.038, Additional file 1: Figure S1).

Because *DNMT3A* mutation was closely associated with *SF3B1* mutation, a good prognostic factor in MDS patients [35, 36], we divided the whole cohort to two subgroups, *SF3B1*-mutated and *SF3B1*-wild type, to evaluate the prognostic significance of *DNMT3A* mutation independent of *SF3B1* mutation. In the *SF3B1-*wild patients, *DNMT3A* mutation predicted worse prognosis (OS, 14.6 ± 4.7 months versus 30.9 ± 3.2 months, *P* = 0.005). On the other hand, in the 48 *SF3B1-*mutated patients, *DNMT3A* mutation had no prognostic implication (OS, 17.7 ± 11.0 months versus 39.7 ± 4.2 months, *P* = 0.858) (Additional file 1: Figure S2).

Intriguingly, the impact of *DNMT3A* mutation on OS and time to leukemia transformation remained significant after adjusting the effects of age, gender, IPSS-R [37, 38], and mutations with prognostic significance in multivariate Cox regression analysis (FAB defined patients: OS: hazard ratio, HR 1.733, 95% CI 1.118–2.688, *P* = 0.014; time to leukemia transformation: HR 3.088, 95% CI 1.574–6.056, *P* = 0.001; 2016 WHO classification defined patients: OS: HR 1.800, 95% CI 1.080–3.000, *P* = 0.024; time to leukemia transformation: HR 2.360, 95% CI 1.129–4.933, *P* = 0.022; Table 3).

**Table 3:** Multivariate analysis (Cox regression) for the overall survival and time to leukemia transformation in MDS patients

	Overall survival		Time to leukemia transformation	
Variable	HR (95% CI)	*P* value*	HR (95% CI)	*P* value*
FAB-defined MDS patients				
Age > 65	1.512 (1.134–2.016)	0.005	0.656 (0.417–1.031)	0.067
Male vs female	1.104 (0.818–1.491)	0.517	0.967 (0.615–1.521)	0.886
IPSS-R higher risk^#^	3.239 (2.199–4.772)	< 0.001	5.258 (2.639–10.477)	< 0.001
*DNMT3A* mutation	1.733 (1.118–2.688)	0.014	3.088 (1.574–6.056)	0.001
*ASXL1* mutation	2.010 (1.434–2.818)	< 0.001	3.396 (2.075–5.556)	< 0.001
*EZH2* mutation	1.019 (0.597–1.741)	0.945	0.885 (0.416–1.880)	0.750
*TET2* mutation	1.416 (0.974–2.059)	0.068	1.398 (0.739–2.644)	0.303
*RUNX1* mutation	1.134 (0.773–1.663)	0.519	1.751 (1.026–2.987)	0.040
*SF3B1* mutation	1.071 (0.679–1.691)	0.767	1.392 (0.699–2.771)	0.347
*TP53* mutation	8.254 (5.338–12.762)	< 0.001	6.653 (3.183–13.909)	< 0.001
2016 WHO-defined MDS patients				
Age > 65	1.649 (1.173–2.319)	0.004	0.731 (0.444–1.203)	0.217
Male vs female	1.184 (0.822–1.705)	0.364	0.988 (0.596–1.637)	0.963
IPSS-R higher risk^#^	3.840 (2.432–6.063)	< 0.001	5.078 (2.390–10.787)	< 0.001
*DNMT3A* mutation	1.800 (1.080–3.000)	0.024	2.360 (1.129–4.933)	0.022
*ASXL1* mutation	1.830 (1.208–2.774)	0.004	3.596 (2.092–6.182)	< 0.001
*EZH2* mutation	1.196 (0.536–2.668)	0.662	0.927 (0.299–2.875)	0.896
*TET2* mutation	1.367 (0.841–2.223)	0.208	1.420 (0.643–3.139)	0.386
*RUNX1* mutation	1.165 (0.723–1.876)	0.531	1.426 (0.753–2.702)	0.276
*SF3B1* mutation	1.251 (0.743–2.153)	0.387	1.423 (0.613–3.305)	0.412
*TP53* mutation	8.517 (5.005–14.492)	< 0.001	8.885 (4.077–19.365)	< 0.001

*Abbreviation: HR*, hazard ratio; *CI*, confidence interval; *IPSS-R*, Revised international prognostic scoring system

*P value < 0.05 was considered significant

^#^IPSS-R higher risk: patients with intermediate, high, and very high risk versus others

### Sequential studies of *DNMT3A* mutations

To investigate the role of *DNMT3A* mutation in clinical evolution, *DNMT3A* gene mutation status was sequentially tested during the clinical course in 431 samples from 148 patients, including 13 patients with *DNMT3A* mutations at diagnosis and 135 patients without the mutation. In the 13 *DNMT3A*-mutated patients, 8 had disease progression, including 6 [unique patient numbers (UPNs) 1, 5, 7, 13, 24, and 37] with AML transformation. Four patients (UPNs 17, 24, 30, and 36) lost the original *DNMT3A* and other concurrent mutations/cytogenetic abnormalities when complete remission (CR) was achieved following curative-intent chemotherapy and/or allogeneic HSCT (Table 4). On the other hand, the other 9 patients with *DNMT3A* mutations at diagnosis retained their mutations during follow-ups. Among the eight with disease progression, one (UPN 37) acquired a novel *RUNX1* mutation when the disease transformed to AML.

**Table 4:** Sequential studies in MDS patients with *DNMT3A* mutations at diagnosis and/or at follow-ups

UPN	Time from diagnosis	Status	Chromosome change	*DNMT3A* mutation (VAF, %)	Other mutations (VAF, %)
1	0 month	MDS-EB2	N	R882H (41.3)	*NRAS* (38.4), *RUNX1* (39), *SF3B1* (38.8)
	6.5 months	AML	ND	R882H (28.5)	*NRAS* (29.1), *RUNX1* (26.7), *SF3B1* (28.3)
5	0 month	MDS-EB2	N	R882H (25.2)	*CEBPA* (23.5)
	5 months	AML	N	R882H (41.8)	*CEBPA* (38.3)
7	0 month	MDS-EB2	N	R882H (34.2)	*RUNX1* (30.3)*, IDH2* (32.8)*, SF3B1* (32.5)
	9 months	AML (s/p C/T)	N	R882H (27.4)	*RUNX1* (28.6)*, IDH2* (27.6)*, SF3B1* (28.3)
10	0 month	MDS-EB1	N	L723TfsX56 (17.1), 1554+1G>T (17)	*U2AF1* (16.5)*, RUNX1* (9.8)
	7 months	MDS-EB1	N	L723TfsX56 (6.3), 1554+1G>T (6.3)	*U2AF1* (6.4), *RUNX1* (3.6)
13	0 month	MDS-EB1	N	R882H (12.4)	*MLL-*PTD
	7 months	AML	+ 8	R882H (20.9)	*MLL-*PTD
	9 months	s/p C/T in CR	N	R882H*	*MLL-*PTD
17	0 month	RAEB-T	N	R882H (35.3)	*IDH2* (7.6), *NPM1* (39.5)
	19.5 months	s/p allo-HSCT	N	–	*–*
21	0 month	RARS	N	Y735C (34.5)	*SF3B1* (44.4), *TET2* (42.3)
	34 months	RARS	N	Y735C (31.1)	*SF3B1* (44), *TET2* (44.3)
23	0 month	RA	N	W313X (45.24), W860R (45.55)	*ASXL1* (35.12)*, IDH2* (48.19)
	3.5 months	CMML	N	W313X (47.74), W860R (44.6)	*ASXL1* (33.31)*, IDH2* (45.29)
24	0 month	MDS-EB1	inv(9)(p11q12)	R882H (8.4)	*NPM1 (7)*
	7.5 months	AML	inv(9)(p11q12)	R882H(33.8)	*NPM1 (31.3)*
	12 months	s/p C/T	inv(9)(p11q12)	–	*–*
	20 months	s/p allo-HSCT	ND	–	*–*
27	0 month	RARS	N	L508SfsX143 (41.1)	*SF3B1 (39.9), TET2* (42.6)
	43.5 months	RARS	N	L508SfsX143 (39.9)	*SF3B1 (39.2), TET2* (41.2)
30	0 month	MDS-EB2	− 7	R882C (30.5)	*U2AF1 (29.2)*
	13.5 months	s/p allo-HSCT	N	–	*–*
36	0 month	MDS-EB1	der(7)t(1;7)(q12;q11),+ 21	R882C (25.4)	*–*
	9.5 months	MDS-EB2	der(7)t(1;7)(q12;q11),+ 21	R882C (19)	*–*
	13.5 months	s/p allo-HSCT	N	–	*–*
37	0 month	RARS	N	R882H (38.2)	*SF3B1 (38.2), TET2 (41.7)*
	7 months	AML	N	R882H (43.4)	*RUNX1 (21.6), SF3B1 (43), TET2 (45.9)*
47	0 month	MDS-EB1	N	–**	*–*
	19 months	MDS-EB1	N	N838D (39.7)	*GNAS (36.5), ASXL1 (17.3), ASXL1 (9), ZRSR2 (6)*

The data of patients who were sequentially studied but had no *DNMT3A* mutation at both diagnosis and follow-ups are not shown

*Abbreviations*: *UPN* unique patient number; −, negative; +, positive; *RA*, refractory anemia; *RARS*, refractory anemia with ring sideroblasts; *RAEB*, refractory anemia with excess blasts; *RAEB-T*, refractory anemia with excess blasts in transformation; *CMML*, chronic myelomonocytic leukemia; *MDS-EB1*, MDS with excess blasts-1; *MDS-EB2*, MDS with excess blasts-2, s/p, status post; *allo-HSCT*, allogeneic-hematopoietic stem cell transplantation; *AML*, acute myeloid leukemia; *C/T*, chemotherapy; *N*, normal karyotype; *ND*, no data; *PTD*, partial tandem duplication; *VAF*, variant allele frequency

*In this sample, *DNMT3A* mutation was not detected by direct sequencing, but 1 of 23 clones showed *DNMT3A* mutation by TA cloning technique. The disease of this patient relapsed at the 10th month from diagnosis (BM blast 31.6%), and he died of AML at 14th month from diagnosis

**No *DNMT3A* mutation was detected by either direct sequencing or TA cloning procedure in this case

Among the 135 patients without *DNMT3A* mutation at diagnosis, 1 (0.7%) patient (UPN 47) acquired a novel *DNMT3A* mutation during sequential follow-up. This patient had MDS with excess blasts-1 (MDS-EB1) at diagnosis when no *DNMT3A* mutation was detectable even using more sensitive cloning method and next generation sequencing. He acquired *GNAS*, *ASXL1*, and *ZRSR2* mutations in addition to *DNMT3A* mutation in the 19th month and died of progressive cytopenia in the 29th month.

We further analyzed the variant allele frequencies of the mutations in the 48 *DNMT3A-*mutated patients by NGS (Table 4). The mutant burden of *DNMT3A* mutations at diagnosis ranged from 8.4 to 45.24% with a median of 31.1%. Among the 13 patients with serial studies during the clinical courses, the mutation burden at subsequent follow-ups, compared to that at diagnosis, was increased in 3 patient (UPNs 5, 13, and 24), decreased in 6 patients (UPNs 1, 7, 10, 17, 30, and 36, Table 4) and stationary in 4 patients (UPNs 21, 23, 27, and 37). All of the three patients with increased *DNMT3A* mutation burden had leukemia transformation. Their variant allele frequencies of *DNMT3A* and other co-occurring mutations were increased at least 10% (10.0–347.1%) at leukemia transformation compared with those at baseline. The patient (UPN 37) who had least increase in variant allele frequency of *DNMT3A* mutation during disease progression acquired *RUNX1* mutation at leukemia transformation. In contrast, the variant allele frequencies of *DNMT3A* and other concurrent mutations were relative stationary or even decreased during follow-up in the patients without leukemia transformation.

## Discussion

In the present study, we identified 19 different *DNMT3A* mutations in 37 (7.9%) of the 469 FAB-defined and 7.7% of the 2016 WHO-defined MDS patients. Similar to previous studies on AML or MDS cohorts [7–10, 12, 13, 17], most mutations are located in the MTase domain, especially at amino acid R882 locus. Of these 19 mutations, 10 are frameshift and 2 are nonsense mutations. They generate truncated peptides with complete or partial deletion of the MTase and are expected to abolish the normal function of *DNMT3A* gene. The R882 mutations result in impaired gene function [7, 39], but the influence of the remaining missense mutations on the enzyme activity are unclear. In this study, the prevalence of *DNMT3A* mutation is 7.9 and 7.7% in MDS according to the FAB and 2016 WHO classification, respectively (Table 1), similar to most of the previous reports (7.8 to 10%) [12, 40–42] but higher than that of Thol et al. (2.6%) [13].

The reports with detailed demographics of MDS patients with *DNMT3A* mutation in literature are limited. In the report of Walter et al., but not in the current study and other studies [40, 42], *DNMT3A* mutations were associated with older age; in contrast, *DNMT3A* mutations were associated with higher platelet count in our study but not in other studies [12, 40, 42]. The association of *DNMT3A* mutations with higher platelet count was also shown in AML in previous studies [8, 9]. No comparison of age and hemogram between patients with and without *DNMT3A* was done in the study of Thol et al. [13] in which only five patients were found to have *DNMT3A* mutation. The causes of differences in the incidence of *DNMT3A* mutation and the clinical characteristics of *DNMT3A*-mutated patients might result from the differences in patient population recruited, detection platform used, sample size, and *DNMT3A* regions screened. In the study of Thol et al. [13], exons 15-23 instead of exons 2-23 of *DNMT3A* gene were analyzed in most patients (173 of 193 patients). Therefore, some patients harboring *DNMT3A* mutations might not be detected, and this might partially explain the lower incidence of *DNMT3A* mutation in their cohort (2.6%).

In this study, *DNMT3A* mutations were positively associated with *IDH2* and *SF3B1* mutations (Table 2). The close association of *DNMT3A* and *IDH2* mutations was also shown in AML [9]. Mutations of *DNMT3A* and *SF3B1*, a component of spliceosome complex frequently mutated in RARS, have been reported to occur concurrently more often than expected by chance in lower-risk MDS patients [17]. In our cohort, the positive association of these two genetic alterations could also be found in lower-risk MDS patients (*P* < 0.001; Table 2). In addition, we could find a trend of positive correlation between these two mutations in higher-risk MDS patients (*P* = 0.098; Table 2). The close associations between *DNMT3A* mutation and RARS and between *DNMT3A* and *SF3B1* mutations in this study (Table 1) might be related with each other. To investigate the associations among the RARS subtype, *DNMT3A* mutation, and *SF3B1* mutation, we divided the whole cohort to RARS and non-RARS patients. The close association of *DNMT3A* and *SF3B1* mutations retained in both subgroups. In contrast, no association between *DNMT3A* mutation and RARS subtype was found when we divided the whole population to *SF3B1*-mutated and *SF3B1* wild-type patients. In the studies of more than 100 genes by high-throughput DNA sequencing, Haferlach et al. [43] and Papaemmanuil et al. [44] also found a positive correlation between *DNMT3A* and *SF3B1* mutations, indicating that interaction between these two gene mutations may play a role in the pathogenesis of MDS, but further investigations are needed to elucidate its mechanism, especially in RARS subtype. No data regarding the association between *DNMT3A* mutation and RARS were shown in these two studies.

*DNMT3A* mutation has been identified as a poor prognostic factor in AML patients [7–11]. However, its prognostic impact on MDS patients remains uncertain. Walter et al. demonstrated *DNMT3A* mutations were associated with shorter survival and higher risk of leukemia transformation in univariate analysis [12], and Thol et al. also reported a higher rate of transformation to AML in patients with this mutation [13]. However, three other studies did not reveal significant impact of *DNMT3A* mutations on survival [15, 17, 44]. In this study, we showed that *DNMT3A* mutation was associated with poor outcomes, including higher risk of AML transformation and shorter OS. Bejar et al. [17] had speculated that the negative prognostic effect of *DNMT3A* mutation might be mitigated by the co-existence of *SF3B1* mutation. In their cohort, 22% patients had *SF3B1* mutation and they did not find the prognostic significance of *DNMT3A* mutation. The same was also true in another study, in which 24% of patients had *SF3B1* mutation [44]. Both cohorts had significantly higher incidence of *SF3B1* mutation than ours (10.2%). It may be possible that *DNMT3A* mutation would have prognostic effect only in MDS cohorts with low prevalence of *SF3B1* mutation. Nevertheless, we distinctly showed that *DNMT3A* mutation was an independent poor prognostic factor for OS irrespective of the status of *SF3B1* mutation and other prognostic factors.

Based on the finding of higher risk of AML transformation and shorter survival in *DNMT3A*-mutated patients, as shown in current study, it would be interesting to investigate the effect of allogenic HSCT in these patients. We found that patients with *DNMT3A* mutations had a better OS if they received allogenic HSCT than those who did not. It implied that HSCT might ameliorate the poor survival impact of the adverse-risk genotype. Further prospective studies with more patients recruited are needed to verify this point. In a study of 46 decitabine-treated AML patients, Metzeler proposed that *DNMT3A-*mutated patients might have better treatment response and longer OS [45]. Subsequently, Traina et al. reported *DNMT3A* mutation as an independent predictor of better response and improved progression-free survival in MDS patients treated with DNMT inhibitors [41]. In our study, only 2 of 36 patients treated with HMA had *DNMT3A* mutation. These two patients had treatment response and OS similar to others. The influence of *DNMT3A* mutation on the treatment response to DNMT inhibitors was not evaluated because of the small number of *DNMT3A*-mutated patients.

*DNMT3A* mutation was found quite stable during disease evolution in AML patients [9, 46], but to the best of our knowledge, the dynamic change of this mutation in MDS patients has not been reported yet in literature. Here we showed that *DNMT3A* mutation was also quite stable in the clinical course of MDS patients; all *DNMT3A*-mutated patients retained the original mutations during sequential follow-ups unless CR was achieved after allogeneic HSCT or intensive chemotherapy. On the other hand, *DNMT3A* mutation was rarely acquired during disease evolution; only one (0.7%) of the 145 *DNMT3A*-wild patients acquired the mutation subsequently (Table 4).

It is well known that age-related clonal hematopoiesis is associated with increase in the risk of hematologic cancer and the majority of the variants occurred in three genes: *DNMT3A*, *TET2*, and *ASXL1* [47–49]. Hematologic cancers were more common in persons with a variant allele fraction of 0.10 or greater. Therefore, it was proposed that *DNMT3A* mutation is relevant for initiating hematopoietic stem cell clonal expansion and an early initiation event for hematological malignancies. Our finding that *DNMT3A* mutation was retained unless CR was achieved was consistent with this hypothesis. In patients who failed to achieve remission, the clone harboring *DNMT3A* mutation survived and may contribute to subsequent relapse. Persistence of *DNMT3A* mutation in some AML patients in CR was described by us and other researchers [9, 50–54]. In a recent study of Gaidzik et al., *DNMT3A* mutant transcript levels in CR did not predict outcome in AML patients [54]. In contrast, Thol et al. showed that patients with *DNMT3A*-mutated lympho-myeloid clonal hematopoiesis (LM-CH) in CR had a higher cumulative incidence of relapse at 10 years compared with those without *DNMT3A*-mutated LM-CH (75 versus 27%) [55]. In the present study, we aimed to delineate the dynamic pattern of *DNMT3A* mutation in MDS development and progression. By NGS, the only patient (UPN 13) who retained his original *DNMT3A* mutation after high intensity chemotherapy finally relapsed. On the other hand, none of the patients in CR who lost their original *DNMT3A* mutation after allogeneic HSCT experienced disease relapse. Our data suggested that *DNMT3A* mutation might be used to assess the treatment response and the risk of relapse after curative-intent treatments in MDS patients. Together, whether retaining of *DNMT3A* mutations after curative-intent treatment is informative for the assessment of the relapse risk in MDS patients remains unclear. It should be cautious to interpret in clinical decision-making and more large-scale studies in MDS patients are warranted to clarify this point.

## Conclusions

We identified associations of *DNMT3A* mutations with distinct clinical features and mutations of *SF3B1* and *IDH2* genes. In addition, we demonstrated that *DNMT3A* mutations independently predicted poor outcomes and were stable in the clinical course. It may be used as a biomarker to monitor the response after curative-intent treatment. Additional file 1, is available at Clinical Epigenetics’ website.

## Additional file

Additional file 1: **Table S1.** The mutation patterns in 37 MDS patients with *DNMT3A* mutations at diagnosis. **Table S2.** Cytogenetics between MDS patients with and without *DNMT3A* mutation. **Figure S1**. Kaplan–Meier survival curves for overall survival among patients with *DNMT3A* mutations stratified by whether receiving allogeneic HSCT or not. **Figure S2.** Kaplan-Meier curves stratified by the status of *DNMT3A* mutations for overall survival among the 421 *SF3B1*-wild type MDS patients (A) and among the 48 *SF3B1*-mutated MDS patients (B). (DOC 242 kb)

## Abbreviations

AML: Acute myeloid leukemia
BM: Bone marrow
CMML: Chronic myelomonocytic leukemia
CR: Complete remission
FAB: French-American-British
HMA: Hypomethylating agents
HSCT: Hematopoietic stem cell transplantation
IPSS: International prognostic scoring system
IPSS-R: Revised international prognostic scoring system
MDS: Myelodysplastic syndrome
MDS-EB1: Myelodysplastic syndrome with excess blasts-1
MDS-MLD: Myelodysplastic syndrome with multilineage dysplasia
NGS: Next generation sequencing
NTUH: National Taiwan University Hospital
OS: Overall survival
RA: Refractory anemia
RAEB: Refractory anemia with excess blasts
RAEB-T: Refractory anemia with excess blasts in transformation
RARS: Refractory anemia with ring sideroblasts
UPN: Unique patient number
