# Supplementary material for: Dynamics of DNMT3A mutation and prognostic relevance in patients with primary myelodysplastic syndrome
Source: Clin Epigenetics. 2018 Apr 2;10:42. doi: 10.1186/s13148-018-0476-1 (PMC5879939; doi:10.1186/s13148-018-0476-1)
Supplement: Supplementary file 1 — Table S1. The mutation patterns in 37 MDS patients with DNMT3A mutations at diagnosis. Table S2. Cytogenetics between MDS patients with and without DNMT3A mutation. Figure S1. Kaplan–Meier survival curves for overall survival among patients with DNMT3A mutations stratified by whether receiving allogeneic HSCT or not. Figure S2. Kaplan-Meier curves stratified by the status of DNMT3A mutations for overall survival among the 421 SF3B1-wild type MDS patients (A) and among the 48 SF3B1-mutated MDS patients (B). (DOC 242 kb) [file 13148_2018_476_MOESM1_ESM.doc]

**Additional file 1**

**Table S1 The mutation patterns in 37 MDS patients with *DNMT3A*** mutations at diagnosis

| **UPN** | **Age/Sex** | | **FAB** | | **Karyotype** | | ***DNMT3A* mutation** | | | | **Other accompanied gene mutations*** |
| --- | --- | --- | --- | --- | --- | --- | --- | --- | --- | --- | --- |
|  |  |  |  |  |  |  | Location | | n.t. change | a.a. change |  |
| 1 | | 60/F | | RAEB | N | Exon23 | | c.2645G>A | | R882H | *NRAS, RUNX1, SF3B1* |
| 2 | | 82/F | | CMMoL | +8 | Exon14 | | c.1627G>T | | G543C | *NRAS* |
| 3 | | 70/M | | RAEB | cplx with 5q-,-7 | Exon14 | | c.1582_1595del | | Y528AfsX119 | *TP53* |
| 4 | | 55/F | | RAEB | N | Exon23 | | c.2645G>A | | R882H | *-* |
| 5 | | 75/M | | RAEB | N | Exon23 | | c.2645G>A | | R882H | *-* |
| 6 | | 75/M | | RARS | -Y | Exon12 | | c.1429+1G>A | | E477DfsX206 | *TET2, SF3B1* |
| 7 | | 75/M | | RAEB | N | Exon23 | | c.2645G>A | | R882H | *RUNX1, IDH2, SF3B1* |
| 8 | | 55/F | | CMMoL | N | Exon21 | | c.2478+1G>A | | F827VfsX6 | *-* |
| 9 | | 77/F | | RAEB | N | Exon14 | | c.1627G>T | | G543C | *RUNX1, SRSF2* |
| 10 | | 70/M | | RAEB | N | Exon18 | | c.2166_2167insA | | L723TfsX11 | *RUNX1, U2AF1* |
| 11 | | 70/F | | RARS | N | Exon23 | | c.2706_2722del | | F902LfsX13 | *SF3B1* |
| 12 | | 78/M | | RA | N | Exon18 | | c.2127delC | | C710AfsX69 | *SRSF2* |
| 13 | | 50/M | | RAEB | +8 | Exon23 | | c.2645G>A | | R882H | *MLL-*PTD |
| 14 | | 53/M | | RAEB-T | -16 | Exon18  Exon23 | | c.2159G>A  c.2644C>T | | R720H,  R882C | *IDH2, ASXL1* |
| 15 | | 72/M | | RAEB | +13,17p- | Exon23 | | c.2644C>T | | R882C | *KRAS, ASXL1, SRSF2* |
| 16 | | 84/F | | CMMoL | der(18)t(9;18)(p12;p11) | Exon14 | | c.1612_1613delAC | | T538HfsX7 | *TET2, SF3B1* |
| 17 | | 35/F | | RAEB-T | N | Exon23 | | c.2645G>A | | R882H | *IDH2, NPM1* |
| 18 | | 62/M | | RAEB-T | N | Exon23 | | c.2644C>T | | R882C | *IDH1, ASXL1* |
| 19 | | 61/M | | RARS | N | Exon23 | | c.2645G>A | | R882H | *SF3B1* |
| 20 | | 84/M | | RAEB | cplx, with -7 | Exon23 | | c.2644C>A | | R882S | *IDH2* |
| 21 | | 78/M | | RARS | N | Exon19 | | c.2204A>G | | Y735C | *TET2, SF3B1* |
| 22 | | 69/F | | RAEB-T | cplx with 5q-,-7 | Exon20 | | c.2385G>A | | W795X | *TP53* |
| 23 | | 57/M | | RA | N | Exon8  Exon22 | | c.939G>A  c.2578T>C | | W313X  W860R | *ASXL1, IDH2* |
| 24 | | 48/F | | RAEB | N | Exon23 | | c.2645G>A | | R882H | *NPM1* |
| 25 | | 55/M | | RAEB-T | ND | Exon23 | | c.2645G>A | | R882H | *MLL-*PTD*, IDH2, SRSF2* |
| 26 | | 86/F | | RA | N | Exon23 | | c.2644C>T | | R882C | *TET2* |
| 27 | | 78/M | | RARS | N | Exon13 | | c.1522delC | | L508SfsX143 | *TET2, SF3B1* |
| 28 | | 59/M | | RAEB | N | Exon23 | | c.2644C>T | | R882C | *RUNX1, TET2* |
| 29 | | 72/M | | RAEB | t(13;21;17)(q14;q22;q11) | Exon23 | | c.2645G>A | | R882H | *ASXL1, RUNX1, SRSF2* |
| 30 | | 45/M | | RAEB | -7 | Exon23 | | c.2644C>T | | R882C | *U2AF1* |
| 31 | | 54/M | | RAEB | cplx, -5,-7 | Exon19 | | c.2204A>G | | Y735C | *TP53* |
| 32 | | 85/F | | RAEB | cplx, -5,-7 | Exon22 | | c.2529delC | | D845TfsX8 | *RUNX1, TP53* |
| 33 | | 89/M | | RAEB | N | Exon8 | | c.889delT | | W297GfsX19 | *SF3B1* |
| 34 | | 72/F | | CMMoL | der(7)t(1;7)(q12;q11) | Exon23 | | c.2644C>T | | R882C | *IDH2* |
| 35 | | 62/F | | RA | ND | Exon19 | | c.2206C>T | | R736C | *SF3B1* |
| 36 | | 42/F | | RAEB | der(7)t(1;7)(q12;q11),+21 | Exon23 | | c.2644C>T | | R882C | *-* |
| 37 | | 54/F | | RARS | N | Exon23 | | c.2645G>A | | R882H | *TET2, SF3B1* |
| **Patients with DNMT3A mutations of uncertain significance (n=9)** | | | | | | | | | | | |
| 38 | | 78/F | | RA | N | Exon15 | | c.1904G>A | | R635Q | *-* |
| 39 | | 76/F | | RAEB | N | Exon18 | | c.2120G>A | | G707D | *RUNX1* |
| 40 | | 74/M | | CMMoL | N | Exon2 | | c.28G>A | | G10R | *-* |
| 41 | | 65/F | | RA | N | Exon21 | | c.2432A>G | | D811G | *-* |
| 42 | | 77/M | | RA | N | Exon13 | | c.1528G>A | | V510I | *ASXL1, IDH2* |
| 43 | | 71/M | | RARS | N | Exon20 | | c.2395C>A | | P799T | *-* |
| 44 | | 84/F | | RAEB | N | Exon18 | | c.2159G>A | | R720H | *-* |
| 45 | | 80/M | | RAEB | inv(3)(q21;q26.2) | Exon17 | | c.1957T>G | | L653V | *JAK2* |
| 46 | | 84/F | | RARS | N | Exon18 | | c.2099C>T | | P700L | *-* |

Nucleotide numberings are according to the National Center for Biotechnology Information reference sequence NM_024426.

*The gene alterations studies included *FLT3*-ITD, *MLL-*PTD, and mutations of *JAK2, K-RAS, N-RAS, RUNX1, ASXL1, EZH2, TET2, IDH1, IDH2, PTPN11, NPM1, WT1, SETBP1, SF3B1, SRSF2, and U2AF1.*

Abbreviations: UPN, unique patient number; FAB, French-American-British; n.t., nucleotide; a.a., amino acid; RA, refractory anemia; RARS, refractory anemia with ring sideroblasts; RAEB, refractory anemia with excess blasts; RAEB-T, refractory anemia with excess blasts in transformation; CMMoL, chronic myelomonocytic leukemia; del, deletion; fs, frameshift; ITD, internal tandem duplication; TKD, tyrosine kinase domain; and PTD, partial tandem duplication; N, normal karyotype; cplx, complex; ND, no data.

**Table S2 Cytogenetics between MDS patients with and without *DNMT3A*** mutation

| **Variables** | **Total**  **(n=437)** | ***DNMT3A* Mutated**^#^  **(n=35, 8.0%)** | ***DNMT3A* Wild**^#^  **(n=402, 92.0%)** | ***P* value** |
| --- | --- | --- | --- | --- |
| **Karyotype group** |  |  |  | 0.787 |
| Normal karyotype | 244(55.8) | 19(54.3) | 225(56.0) | 0.861 |
| Loss Y* | 6(1.4) | 1(2.9) | 5(1.3) | 0.396 |
| Del5q/Monosomy 5* | 2(0.4) | 0(0) | 2(0.5) | >0.999 |
| Del 20q* | 11(2.5) | 0(0) | 11(2.7) | >0.999 |
| Trisomy 8* | 23(5.3) | 2(5.7) | 21(5.2) | 0.705 |
| Del7q/Monosomy 7* | 17(3.9) | 2(5.7) | 15(3.7) | 0.637 |
| Other abnormalities | 134(30.7) | 11(31.4) | 123(30.6) | >0.999 |

* As the sole abnormality.

^#^ Number of patients (% of patients in this subgroup among patients with or without *DNMT3A* mutation)

**Figure Legends**

**Figure S1**

Kaplan–Meier survival curves for overall survival among patients with DNMT3A mutations stratified by whether receiving allogeneic HSCT or not

**Figure S2**

Kaplan-Meier curves stratified by the status of *DNMT3A* mutations for overall survival among the 421 *SF3B1*-wild type MDS patients (A) and among the 48 *SF3B1*-mutated MDS patients (B).

**Figure S1**


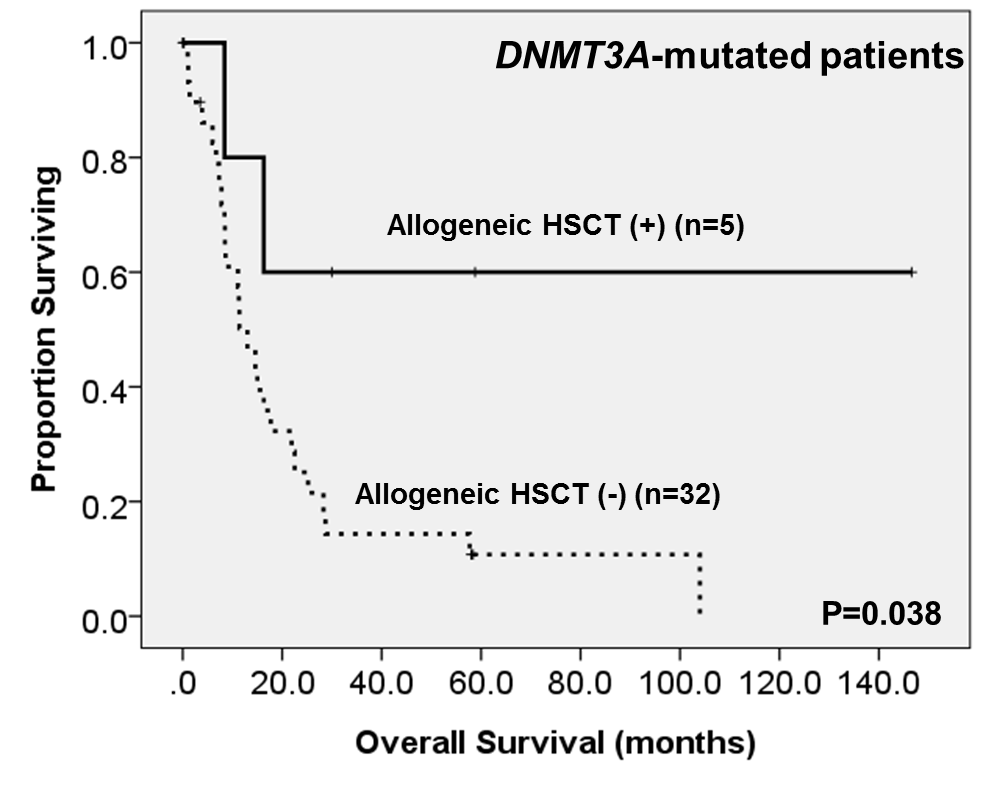


**Figure S2A**


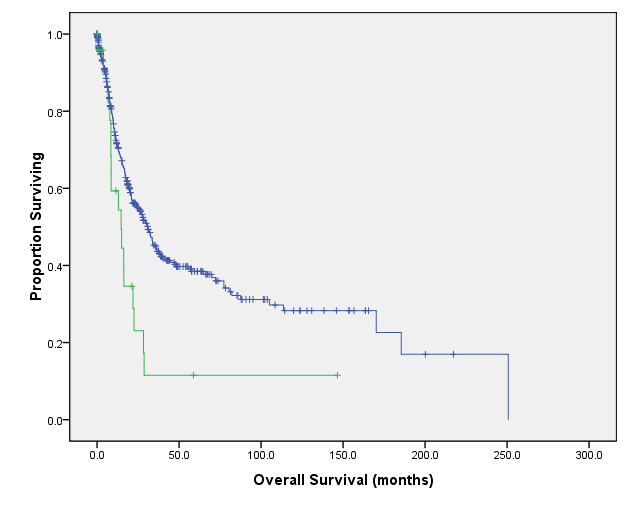


***SF3B1*-wild type patients**

*DNMT3A*-mutated

*DNMT3A*-wild type

P=0.005

**Figure S2B**


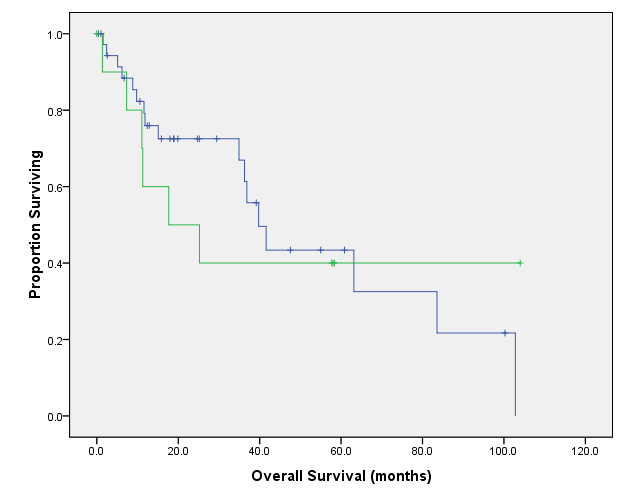


***SF3B1*-mutated patients**

P=0.858

*DNMT3A*-mutated

*DNMT3A*-wild type
